# Integrated Analysis of Species Invasion Under the Telecoupling Framework: Burmese Python Across Southeast Asia, the USA, and Beyond

**DOI:** 10.3390/biology15171494

**Published:** 2026-09-02

**Authors:** Camille Klemann, Jianguo Liu

**Affiliations:** Center for Systems Integration and Sustainability, Department of Fisheries and Wildlife, Michigan State University, East Lansing, MI 48823, USA; klemannc@msu.edu

**Keywords:** invasive species, coupled human and natural systems, sustainability, *Python bivittatus*

## Abstract

In an increasingly globalizing world, invasive species have become a serious concern worldwide. Numerous studies have focused on the biology of different invasive species, but few have systematically integrated socioeconomic and environmental factors. The framework of telecoupling (socioeconomic–environmental interactions over distances) can help address this challenge by allowing researchers to integrate human-nature interactions across distances. To demonstrate this potential, the framework was applied to the invasion of the Burmese python (*Python bivittatus*) from Southeast Asia. This species is a large constrictor currently invading the Greater Everglades ecosystem in South Florida, USA, through the pet trade. Results show many causes and effects of the invasion. The analysis also exposes several research gaps. The most prevalent is a lack of published research about the species’ native range, including biology in the wild as well as a socioeconomic understanding of the people involved in and affected by the invasion. The telecoupling framework connects scattered information, providing insights into flow-centered management approaches that could lead to more effective population control. This analysis shows the telecoupling framework’s potential to improve understanding of the complexities of invasive species, their causes, effects, future research directions, and management strategies.

## Simple Summary

As the world becomes more connected through trade and travel, more plants and animals become invasive species beyond their native habitats. Burmese pythons are a good example of this. Native to Southeast Asia, they were brought to America as pets and later established themselves as an invasive species in southern Florida, where they have caused major ecological impacts. In this review, we use the telecoupling framework, which examines interactions between humans and nature across distant places, to analyze the Burmese python invasion from a global perspective. By linking the native range, invasive range, and intermediary locations, this approach highlights how biological, economic, and social factors interact across regions. Our analysis reveals that most research on this invasion has focused on Florida, while much less attention has been given to the species’ native range, the people involved in the trade, and the broader human–environment interactions that enabled its spread. Thus, major knowledge gaps remain about the species’ native range in Southeast Asia, particularly the ecology of Burmese pythons in the wild. Filling these gaps can enhance the effective management of invasive species worldwide.

## 1. Introduction

More than 37,000 species worldwide inhabit areas outside their native range [1]. This number continues to rise by about 200 per year as the world becomes more interconnected due to modern trade and travel [1,2]. Among species outside their native ranges, more than 3500 are invasive, causing socioeconomic and ecological changes such as declines in species richness and trophic shifts. On average, introducing a single invasive species to an ecosystem results in a 16.6% decrease in total species richness. Furthermore, in terrestrial habitats, total species richness decreases by an average of 27.69% if the invader is a predator [3].

As global interconnectivity increases, the problem of species invasions becomes more relevant to scientists and societies worldwide. To address this problem, it is important to improve our understanding of the interactions between human and natural systems. Many studies focus on the biology and physiology of invasive species [4,5], while others focus on their negative environmental impacts [6,7]. Studies also examine the effects of invasive species on humans. For example, the spread of diseases such as yellow fever and human fasciolosis is caused by the liver fluke *Fasciola hepatica* [8]. More recent studies examine both the economic repercussions of direct ecological damage and the funding required for management efforts [9]. For example, an estimated $120 billion is lost annually in the United States when monetary value is assigned to species extinctions, biodiversity loss, degradation of ecosystem services, and aesthetic impairment [9]. Additionally, there is growing recognition of the need for human dimensions in wildlife conservation [10]. However, previous studies largely focus on either the environmental or the socioeconomic aspects of invasive species without integrating them. Furthermore, these studies often focus on a localized site and fail to examine the full course of a species’ invasion. The telecoupling framework, however, offers a systematic method for investigating socioeconomic and environmental interactions across distances worldwide [11].

Telecouplings are the socioeconomic and environmental interactions that occur across distances. The telecoupling framework classifies each site as a coupled human and natural system, allowing researchers to analyze how humans and nature interact across spatial scales [12]. The framework is hierarchical, with different components at each level. At the telecoupled system level, coupled, interrelated human and natural systems are connected by flows. Then, at the coupled system level, each system has three interrelated components: agents, causes, and effects [11]. This results in five components in total: systems, agents, flows, causes, and effects (Table 1).

Systems are coupled human and natural systems in which humans and nature interact [13]. The systems are connected through various flows such as movement of energy, matter, information, people, and organisms [14]. There are three types of systems: sending, receiving, and spillover. Sending systems are sources from which flows enter the receiving system. Receiving systems, therefore, are the recipient sites of flows. Spillover systems affect and/or are affected by the interactions between the sending and receiving systems. This can occur by acting as an intermediate stopover between the two systems, serving as the path between the sending and receiving systems, or through other interactions with the sending and receiving systems [12]. In the case of invasive species, the sending system is their native range, the receiving system is their invasive range, and spillover systems can be a myriad of situations, one example being transit regions. Causes are the factors that influence the emergence and dynamics of telecoupling. These, in turn, lead to socioeconomic or environmental effects. The flows are central to telecoupling, as they determine whether a system is sending, receiving, or experiencing spillover. Flows can manifest themselves in different ways, but they are broadly defined as the material, energetic, and informational movements between systems [11]. Agents are the autonomous decision-makers that affect flows.

**Figure 1:**
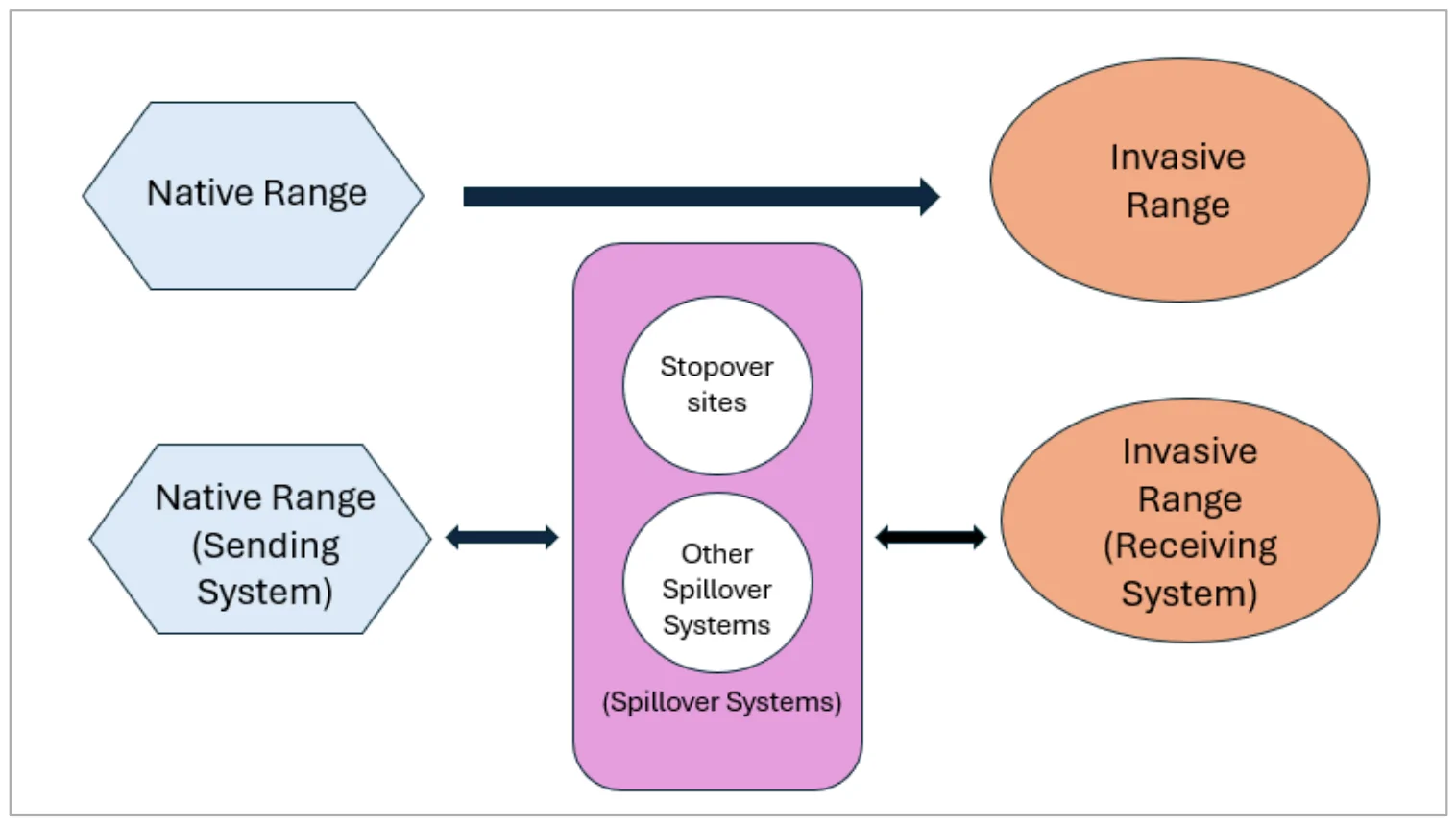
Comparison between traditional invasive species research (**top**) and telecoupling analysis (**bottom**). In traditional research, the unidirectional arrow indicates the flow of species from the native range to the invasive range. In telecoupling analysis, bidirectional arrows indicate flows not only of species but also of energy, information, capital, people, etc., between the native and invasive ranges. Furthermore, native and invasive ranges are considered coupled human and natural systems. Another type of system, spillover systems (such as stop sites of species moving from the sending to receiving systems) that affect or are affected by the flows between sending and receiving systems, is also integrated. Each system has three major components (causes, agents, and effects).

The framework has been used to analyze various topics such as international trade [15], migratory species [16], and other invasive species [17]. Applying the telecoupling framework can yield insights, such as identifying research gaps and previously unknown connections across different parts of the world [17] (Figure 1). While the framework has broad utilities, its application to invasive species has been limited.

To demonstrate the potential of this framework in invasive species research, we used the Burmese python (*Python bivittatus*) as an example. The Burmese python is one of the world’s largest snakes. It is native to Southeast Asia but has established itself as an invasive species in southern Florida, USA, since at least 1979 [18]. It preys on a wide variety of native wildlife, contributing to severe declines in mammal populations [19]. Although several management efforts have been attempted, they have been difficult and largely ineffective.

The telecoupling framework helps account for both the biological and human dimensions involved in the Burmese python’s invasion, offering a new perspective that may lead to more effective management efforts. This paper first analyzes the invasion using the telecoupling components and explores novel insights and opportunities. It then discusses research and management gaps and the framework’s applicability to other invasive species. This analysis closes with a discussion of the challenges and opportunities for framework operationalization.

## 2. Applying the Telecoupling Framework to Burmese Python Invasion Research

This section investigates the application of the telecoupling framework to Burmese pythons across the five integrated components: systems, flows, agents, causes, and effects. Sources of information were identified through literature searches in Google Scholar using keywords such as “Burmese python,” “*Python bivittatus*,” “invasive species,” “telecoupling,” and “pet trade.” These keywords were also translated into various languages such as Thai, Vietnamese, Russian, Khmer, Lao, and Bengali to ensure a wide range of sources. Studies were then chosen based on their applicability to each telecoupling component, their publication date, and their affiliations (affiliations with accredited universities or government agencies were favored). Searches were conducted from 10–20 September 2025; 11, 13, 20–22 January 2026; and 16–20 March 2026.

This section begins with an overview of Burmese python biology, followed by a summary of their invasion of the South Florida Greater Everglades ecosystem. In the case of the Burmese pythons, systems refer to their native range (sending system) and the Greater Everglades ecosystem (receiving system). The sites between them, such as stopover sites for international pet traders and the homes of American pet owners, are examples of spillover systems. Agents include the Burmese pythons themselves, government agencies, and private citizens such as pet Burmese python owners and the public. These alter the flow of energy, materials, money, and/or information between and within the systems. The invasion of Burmese pythons into the Greater Everglades Ecosystem is a result of various causes. These include the Burmese python’s natural ability to thrive in the Greater Everglades habitat and human factors such as a cultural inclination to release pet reptiles and more trading of exotic pets between the United States and Southeast Asia. This invasion has many effects, which can be divided into two major categories: socioeconomic and environmental. Socioeconomic effects include a prohibition of the sale of Burmese pythons in Florida and increased regulation of their import into the United States. Environmental effects include decreased biodiversity in the Greater Everglades ecosystem and the rise in invasive parasites. The invasion of Burmese pythons will be further analyzed in the coming sections using the telecoupling framework.

### 2.1. Overview of Burmese Pythons

**Figure 2:**
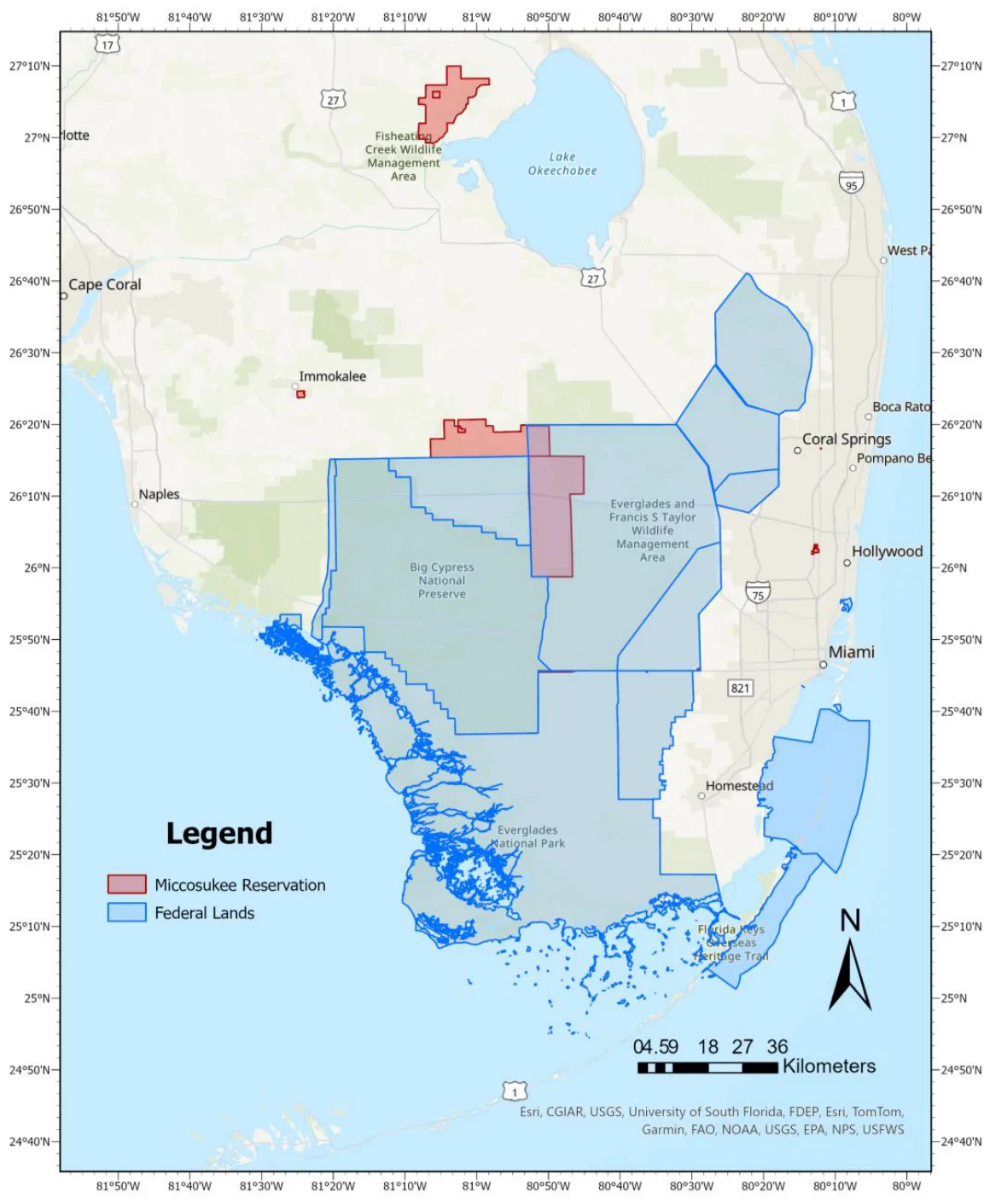
Map of federal lands in the Greater Everglades ecosystem. Highlighted lands include Big Cypress National Preserve (BICY), Everglades National Park (ENP), and tribal lands stewarded by the Miccosukee Tribe of Indians of Florida.

Burmese pythons (*Python bivittatus*) are large nonvenomous constrictors. Their native range is vast, encompassing most of Southeast Asia. They are known to occupy an array of habitat types in their native range. These may include tropical lowlands, grasslands, forests, agricultural land, and aquatic habitats [20]. Along with being able to survive in a wide range of habitat types, they are also able to prey on a variety of wildlife. They have been documented preying on mammals, birds, and reptiles [18]. Their habitat generality, combined with the ample prey, ideal temperatures, and large area, has made them a prolific invasive species in South Florida, USA, specifically in the Greater Everglades ecosystem (Figure 2).

The Greater Everglades ecosystem is a large, shallow watershed. The first reported sighting of a Burmese python in the Everglades was in 1912. The first body of a Burmese python was found there in 1979, but it was not considered a reproducing population until the 1990s and early 2000s [18]. Since then, numerous efforts have aimed to better understand their biology and develop effective control tools. These methods include removal programs, trapping, and radiotelemetry to monitor their social behaviors. However, because the Greater Everglades ecosystem is inaccessible and Burmese pythons are cryptic, it has been difficult to estimate abundance and therefore assess the efficacy of control tools [18].

### 2.2. Systems

**Figure 3:**
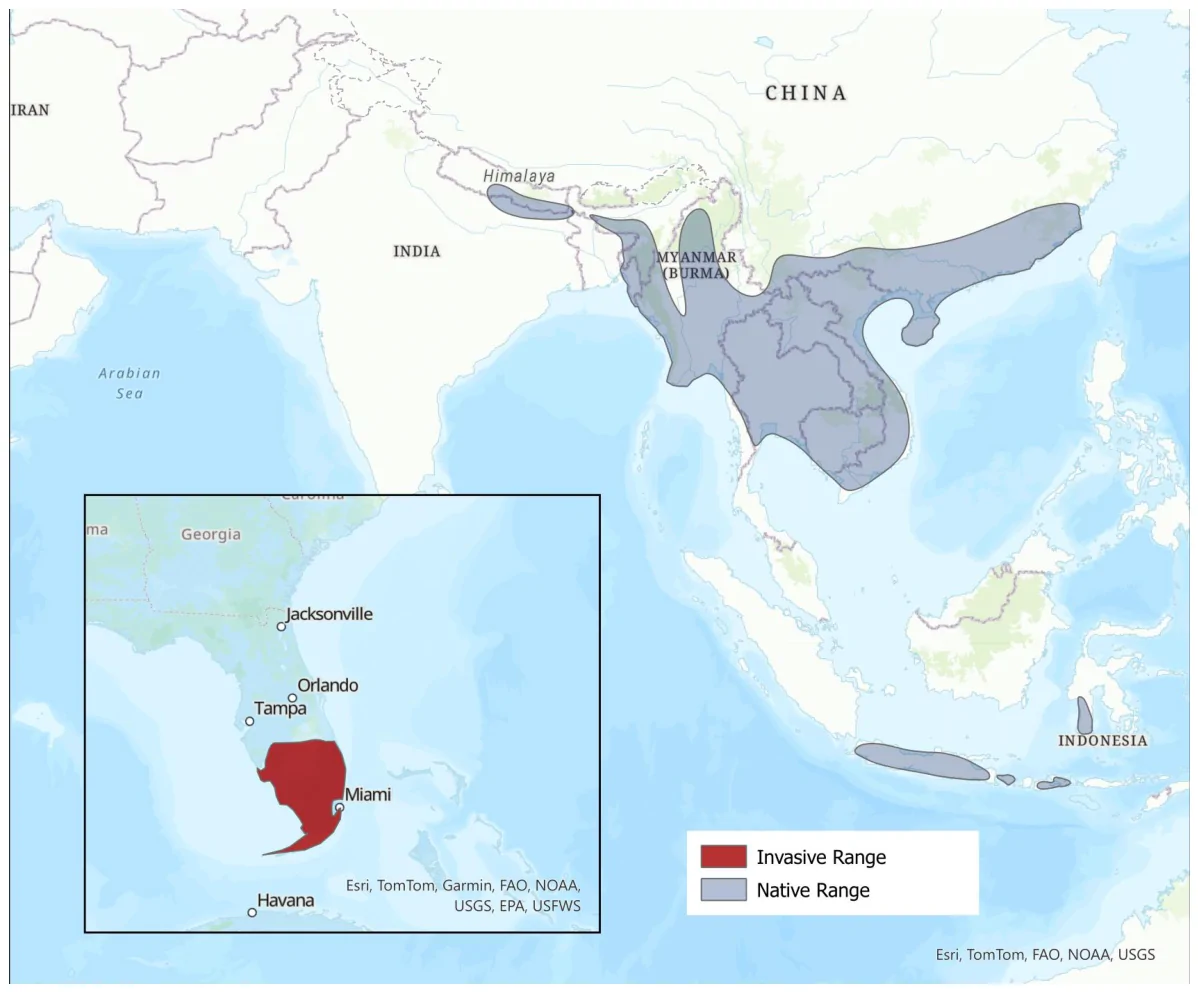
Map of Burmese python native and invasive ranges. This was taken from occurrence points of Burmese pythons from the Global Biodiversity Information Facility spanning the years 1979–2024 [21]. Polygons were then drawn to encompass the occurrence points and visualize their range.

Because Burmese Pythons are an invasive species, three major systems interact with each other. The sending system is their native range, a large area of Southeast Asia, particularly in Bangladesh, Myanmar, Laos, parts of Thailand north of the Isthmus of Kra, Cambodia, Vietnam, and China. There are also disjunct populations in Northern and Northeastern India and Southern Nepal (Figure 3) [20]. The receiving system is Florida, USA, more specifically the Greater Everglades ecosystem (Figure 3). The Greater Everglades ecosystem is 87 km (60 miles) in length and 161 km (100 miles) in width, located on the southernmost tip of the Florida peninsula (Figure 2). It is mainly composed of parks and preserves, including Everglades National Park (ENP), Big Cypress National Preserve (BICY), Collier-Seminole State Park (CSSP), Fakahatchee Strand Preserve State Park (FSPSP), Picayune Strand State Forest (PSSF), Rookery Bay National Estuarine Research Reserve (RBNERR), Biscayne National Park (BISC), and Arthur R. Marshall Loxahatchee National Wildlife Refuge (LNWR), as well as numerous public and private conservation lands and property managed by the South Florida Water Management District and the Florida Fish and Wildlife Conservation Commission. These areas are mostly uninhabited and not easily accessible [18].

Burmese pythons’ native range has a variety of habitat types, including urban habitats. They have been reported entering human settlements and preying on livestock. This leads to human–wildlife conflict in their native range [20]. Conversely, most of their invasive range is uninhabited and publicly owned [18]. In their native range, they have been declining due to direct human persecution and habitat degradation [20]. Their invasive range suffers from a similar problem: abundance is difficult to detect. However, Burmese python removals have sharply increased since the start of removal efforts in the early 2000s [18].

Spillover systems include the shipping locations of Burmese pythons from their native range into the United States. They can also be within the private properties of Burmese python keepers who release(d) their pet pythons into the wild. Because many breeders, shippers, and keepers operate privately, these populations are difficult to quantify. Evidence also suggests smuggling of Burmese pythons into the United States, though this is difficult to record [22]. For legal importation of reptiles into the United States, the U.S. Department of Customs and Border Protection requires importers to report the animals they wish to ship [23]. All imports and exports must go through one of the 18 ports designated to handle all wildlife shipments. These ports are in Anchorage, Atlanta, Baltimore, Boston, Chicago, Dallas/Fort Worth, Houston, Honolulu, Los Angeles, Louisville, Memphis, Miami, New Orleans, New York, Newark, Portland, San Francisco, and Seattle [23]. Therefore, spillover systems can occur in the homes of reptile keepers, not exclusively among keepers who release their pythons into the wild, ports around the United States, and smugglers along the route from Southeast Asia to Florida.

### 2.3. Agents

**Figure 4:**
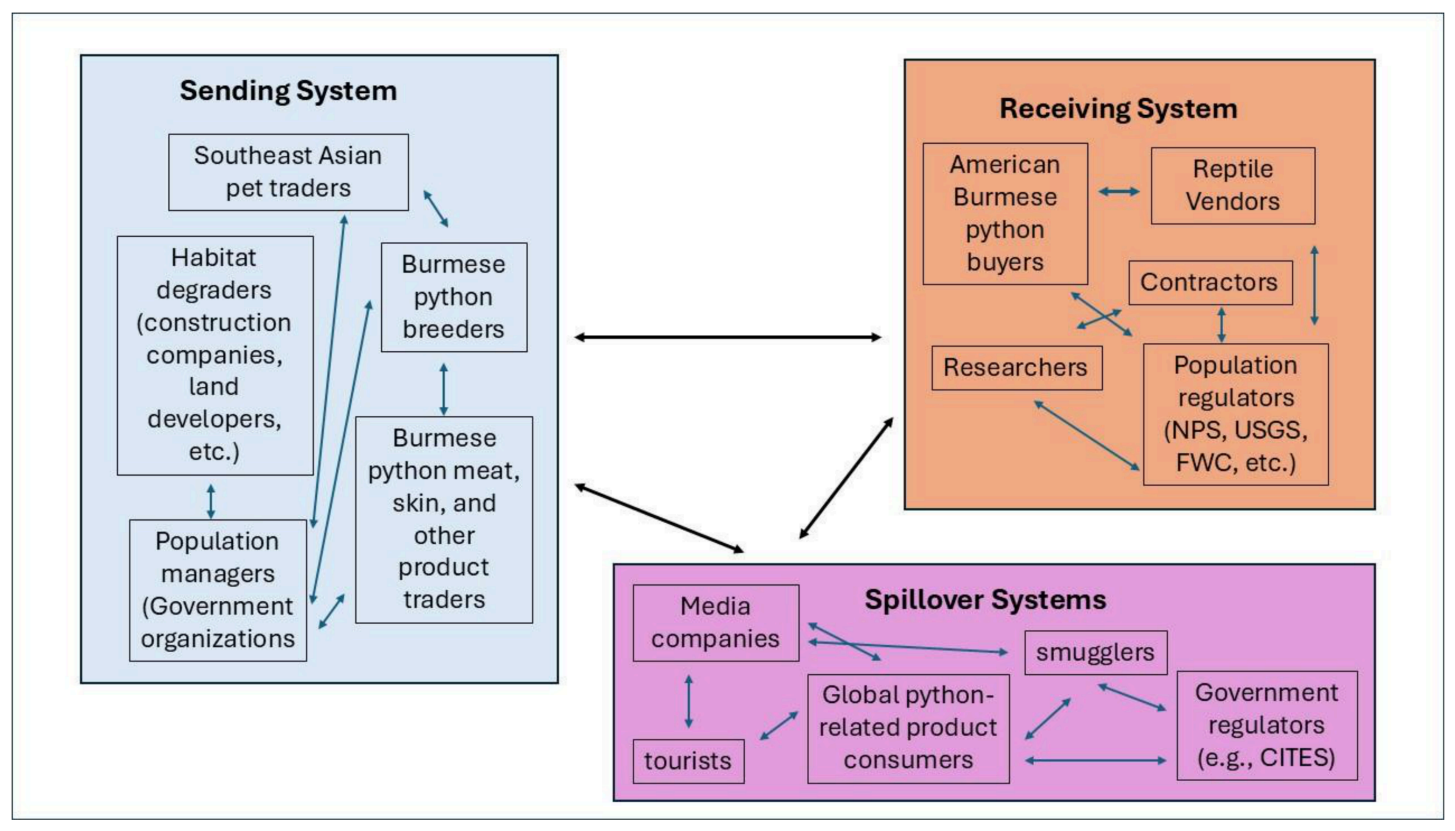
Key agents involved in the movement and invasion of Burmese pythons into South Florida. Blue arrows represent the interactions between agents within a system, while black arrows represent the interactions between the systems.

Given the large spatial distance between the sending and receiving systems, many agents, both directly and indirectly, contribute to the movement of Burmese pythons into South Florida. The first are pet traders in Southeast Asia (Figure 4), who largely contributed to the movement of pythons until around the 1980s. In the 1980s, *National Geographic* published an article about the sale of an albino Burmese python, which led to an explosion in popularity and prompted American breeders to breed them domestically [22]. Reptile-keeping publications, as well as more general publications such as *National Geographic*, also act as agents because they can indirectly increase or decrease the movement of Burmese pythons and of information.

Because of the trade relationship between Southeast Asia and reptile keepers in the United States, many American agents exist (Figure 4). The most obvious are the buyers of Burmese pythons as pets. Pet owners are associated with the beginnings of their invasion in the Greater Everglades ecosystem through releases and escapes. This is a large and general market. Reptile keepers do not have regional biases and are found in urban, suburban, and rural areas. In 2010, 4.7 million homes across the United States owned 13.6 million pet reptiles [23]. A substantial body of writing and media has also changed the flow of Burmese pythons. Sensationalist articles such as the previously mentioned one from *National Geographic* contributed to an interest in keeping Burmese pythons. Now, the media has villainized the Burmese python, especially in South Florida [24]. In a 2014 study of Florida residents, 79% of survey participants said they were likely or very likely to pay attention to a news story about invasive species [25]. Additionally, 73% of respondents indicated they believe there should be stronger restrictions on owning and selling Burmese pythons specifically [25]. This results in media organizations, consumers of reptile-focused information, and the public becoming agents that can change the flow of money, information, and Burmese pythons themselves (Figure 4).

Government organizations also act as agents. As previously mentioned, the U.S. Department of Customs and Border Protection has regulations on wildlife imports. Additionally, the Convention on International Trade in Endangered Species of Wild Fauna and Flora (CITES) partly regulates the legal international trade of giant constrictors, including the Burmese python (Figure 4). Burmese pythons have been kept as pets in the United States since the early 1900s. Their numbers greatly increased by the 1970s, and CITES began to monitor their movement in 1975. Between the late 1970s and 1994, 54% of live Burmese pythons imported into the United States came from Thailand, followed by Myanmar (33%). From 1994 to 2011, however, most imported Burmese pythons came from Vietnam (96%) due to Thailand beginning to enforce its protected status due to declining populations. Finally, in 2012, the Burmese python was added to the injurious species provision of the Lacey Act (a 1990 law created to address the illegal wildlife trade, protect at-risk species, and stop international importation of injurious species). At a more local level, the Florida Fish and Wildlife Conservation Commission (FWC) began regulating Burmese pythons in Florida by adding them to the Reptiles of Concern list. This required owners of Burmese pythons to obtain a license to continue possessing them. They were subsequently regulated over the following decades before being prohibited in Florida in 2021 [18].

Government agencies have also taken a more direct role in moving Burmese pythons by implementing removal efforts using contractors and trained volunteers. Organizations such as the South Florida Water Management District (SFWMD), FWC, and the National Park Service (NPS) began removal in 2017 on United States Department of the Interior lands. As of 31 December 2021, government-funded removal efforts eliminated 13,746 pythons. Government-funded research efforts also conduct smaller-scale removals. These include radiotelemetry tracking to understand the social behavior of Burmese pythons and to remove breeding individuals [18].

Lastly, Burmese pythons themselves act as agents. The initial invasion began not only with releases but also with escapes [18], moving the pythons from their spillover system to their receiving system. Burmese pythons, though docile, grow to large sizes that give them the capacity to kill or injure their owners from severe bites or constriction. Their large size also requires a reptile keeper to provide a large enclosure that is difficult to secure. This allows the python to escape and find its way into the wild [22].

A vast network of agents spans the trade routes from Southeast Asia to South Florida. Southeast Asian ecology, governmental regulation, and human persecution interplay with the United States’ markets, media, and policies, showing the interconnectivity between systems.

### 2.4. Flows

The main material flow between the sending and receiving systems is the pythons themselves, moving from the sending system to the receiving system. They also facilitate an energy flow through their diet within each system. They prey on various vertebrates, most of which are mammals and birds. Records also show Burmese pythons eating lizards, frogs, and other snakes. Within their native range, there have been records of them being consumed by king cobras (*Ophiophagus hannah*) and raptors. In South Florida, documented predators include bobcats (*Lynx rufus*), indigo snakes (*Drymarchon couperi*), Florida cottonmouths (*Agkistrodon conanti*), American alligators (*Alligator mississippiensis*), and American crocodiles (*Crocodylus acutus*) [18].

The more pressing flow now is not Burmese pythons coming from Southeast Asia into the United States; it is their expansion into the Greater Everglades ecosystem. The 1979 report was about a road-killed python on US-41, which forms the northern boundary of Everglades National Park (Figure 2). This is located slightly less than 100 km from the southern tip of the Florida peninsula. Between 2019 and 2021, pythons were reported to occupy most of southern Florida, covering an area of 30,000 square kilometers. Evidence also shows that Burmese pythons are widespread across many terrestrial habitats in Everglades National Park [18].

There are also large flows of information facilitated by many news and media organizations. Burmese pythons have become a well-known issue in South Florida, and there is a widespread negative opinion of them. Within Florida, they are often discussed in terms of being “deadly predators” and “highly dangerous” [24]. 73% of Florida residents replied “yes” when asked whether there should be more restrictions on owning and selling Burmese pythons as pets, and 73% also replied “yes” when asked whether the efforts to control python populations in the wild should be strengthened [25]. It can also be assumed that information flows beyond people who consume python-focused media. This is perhaps best demonstrated by the negative connotative turn Google searches began to take as the python invasion progressed [22]. In a more academic sense, researchers also publish their findings as they study pythons in the wild and/or in captivity. Papers include the 2023 paper by Guzy et al. titled “Burmese pythons in Florida: A synthesis of biology, impacts, and management tools” [18]. Information also flows through tourists as they visit the Greater Everglades ecosystem. In the year 2023, there were over 1.2 million visitors to Everglades National Park and 2.1 million visitors to Big Cypress National Preserve [26].

Additionally, monetary flows between systems play a large role. Breeders or capturers of Burmese pythons in Southeast Asia are paid to ship live snakes to clients worldwide; however, there are costs to shipping. As of 2009, reptile shipments generated an estimated $5 million to $7 million in revenue, and 520,724 pet reptiles were imported into the United States, averaging $10 to $13 per pet [23]. These pythons are either received directly by prospective reptile-keepers or are more likely sent to pet stores. Pet stores realized $17 million to $22 million in revenues in 2009 [23]. Ancillary flows of money are also involved in the care and keeping of pet Burmese pythons. In 2009, there was an estimated $56.5 million to $70.5 million in revenue from the sale of ancillary reptile care products and between $22 million and $25.5 million from breeding rodents and bugs for consumption by pet reptiles [23]. In the same year, an estimated $57.5 million was spent on veterinary expenditures for snakes. Overall, there is a large flow of money involved in keeping reptiles; however, it is currently illegal to own and sell Burmese pythons in the state of Florida. Now, money flows through research and management efforts.

Research expenditures for managing Burmese pythons totaled $10.6 million between 2004 and 2023 [18]. One of the simplest methods of python detection is visual surveys, but they also come at a cost. From 1 May 2019 to 30 April 2021, visual surveys cost $628,471. This includes Fish and Wildlife Commission staff salaries, contractor wages, and material costs. Additionally, research involving radiotelemetry costs an average of $30,880 per breeding season (December to March) [18]. Money also flows through public consumption of media about Burmese pythons and through tourists via more direct methods such as park admission fees. Money also flows indirectly from tourists through purchases of outdoor recreation equipment, food, boarding, and flights.

### 2.5. Causes

Simply put, the invasion of Burmese pythons was caused by pet owners releasing them into the wild. There are several hypotheses about when this occurred, but it is most likely that their establishment in South Florida is from an initial release of a small number in Everglades National Park before 1985 [27]. Evidence also suggests a second introduction northwest of ENP between the late 80s and 90s [18]. Once introduced to the Greater Everglades ecosystem, pythons spread quickly because their physiology is well suited to the environment, providing an ecological basis for their invasion. In their native range, they are found in a wide variety of climates and habitats. The comparatively small area they inhabit in southern Florida does not offer the same diversity; however, this may be an advantage. Burmese pythons are at risk of death at temperatures less than 10 °C, and 5 °C is considered their critical thermal minimum [18]. In ENP, average yearly temperatures range from 12 °C to 33 °C, well above their critical thermal minimum [28]. However, the Florida population also shows adaptation even after a relatively short time scale. The current population has higher metabolic rates than those before a major freeze event in 2010, allowing them to maintain higher body temperatures and survive cooler temperatures [29]. Additionally, they have an energetic advantage due to their large size and ambush-style hunting [30]. Burmese pythons are also dietary generalists. Reports describe them ingesting large vertebrates such as leopards in their native range and white-tailed deer (*Odocoileus virginianus*) in their invasive range. However, they are known to eat organisms as small as cotton mice (*Peromyscus gossypinus*) and shrews [18].

As for their owners, there are many reasons a person might release their pet. Burmese pythons are large constrictors that could pose a physical threat to their owners [22]. Large pets have a higher probability of release, which can increase with higher numbers of imported individuals and lower purchase prices [31]. Between January 2005 and May 2010, Burmese pythons were the second most often imported large constrictors, second only to reticulated pythons (*Malayopython reticulatus*) [23]. Reptiles were the most highly exported taxa from international ports, making up 66% between 1999 and 2013. During the same period, Vietnam was the highest exporter of reptiles, accounting for 96% of Burmese pythons exported from 1994 to 2011 [18,32]. They were also sold at low prices, ranging from $20 at reptile shows to $65–$80 at pet stores [33]. There was also an explosion in the keeping of exotic reptiles in the 1980s and 1990s, in contrast to the mostly native reptiles kept as pets from the 1940s to the 1970s [34].

There are also political, economic, and technological causes. Both politically and economically, the 20th century was a turbulent time for the relationship between Southeast Asia and the United States. Through the 1960s and into the 1970s, America was deeply involved in Southeast Asian affairs, mainly fighting proxy wars against its Cold War rival, the USSR (Union of Soviet Socialist Republics) [35]. America’s main interests were maintaining regional stability and limiting the spread of communism. In the early 1980s, the states in the Association of Southeast Asian Nations (ASEAN) (Indonesia, Malaysia, the Philippines, Thailand, Singapore, and Brunei) made the case to the US that these issues were dependent on economic prosperity [35]. Therefore, it was in both parties’ interest for America to invest in their markets, leading to a boom in trade between the ASEAN states and America. This practice was maintained well into the 1990s. In 1993, Assistant Secretary for East Asian and Pacific Affairs, Winston Lord, promised to promote “American trade and investment” [36]. This intermingling of politics and economics led to a growth in imports from Southeast Asia of all kinds, such as textiles, electronics, and live reptiles.

Technologically, the biggest advance was air travel. During World War II, planes became a way to carry goods around the world [37]. After the terms of surrender were signed in 1945, the public became interested in using airplanes for commercial shipping. By the late fifties, air travel had become significantly faster [38]. This substantial reduction in shipping time increased the chances of survival for live animals [39]. Also, in the 1950s, a packing method for reptiles and amphibians was developed that maintained a steady internal temperature and humidity throughout flight [40]. Over the following decades, air shipping would be refined as air travel became safer, more reliable, and more accessible. This allowed Burmese pythons to be shipped overseas by large distributors in cargo planes and for small sellers to transport them on commercial flights.

### 2.6. Effects

The effects of the Burmese python invasion can be categorized as being ecological or socioeconomic, though these are often interrelated. Ecologically, pythons are proven to directly change the Greater Everglades ecosystem through their diet [41]. They are documented eating state-threatened species like the little blue heron (*Egretta caerulea*), roseate spoonbill (*Platalea ajaja*), and the Big Cypress fox squirrel (*Sciurus niger avicennia*). There is also evidence that they have preyed on federally endangered species such as the Key Largo woodrat (*Neotoma floridana smalli*) and the Key Largo cotton mouse (*Peromyscus gossypinus allapaticola*) [18]. They also threaten species of least concern. Mammals of a range of sizes have declined significantly in abundance from 2003 to 2011. During this period, mammal observations (common species include the raccoon (*Procyon lotor*), Virginia opossum (*Didelphis virginiana*), bobcat, and white-tailed deer) declined by 85–100% [18].

The spread of Burmese pythons also has indirect ecological impacts. In their native range, they host ectoparasites, blood parasites, intestinal parasites, and other types of endoparasites. In southern Florida, they are hosts to native North American parasites such as pentastomes (*Porcephalus crotali*), but they also carry the non-native Asian pentastome, *Rallietiella orientalis.* The non-native Asian pentastome has spilled over into native snake species such as the federally threatened eastern indigo snake. Regarding ectoparasites, Burmese pythons are known to carry non-native tick species (*Amblyomma rotundatum* and *A. dissimile*) that could potentially spread to native wildlife in South Florida [18].

Another indirect consequence of the Burmese python invasion is changes in trophic structure, leading to trophic cascades [42]. Heavy predation on mammals has increased more fecund species, such as those in the order Rodentia, while larger, more specialized species such as bobcats are more vulnerable. This increase in rodent populations has changed disease spread and may alter processes such as seed dispersal, scavenging, and nutrient cycling. Their predation of white-tailed deer has lessened the abundance of this important herbivorous species, leading to changes in vegetation dynamics [18].

The movement of Burmese pythons into the Greater Everglades ecosystem has also had socioeconomic effects. They have been labeled as a problem species, leading to changes in public perception and policy actions [20]. Between 2012 and 2021, Florida enacted laws to further restrict the import, sale, and possession of live Burmese pythons, culminating in a 2021 prohibition [18]. Additionally, the growing understanding of the invasion’s severity spurred research into Burmese python biology and sparked a conversation about the ethics of the exotic pet trade. This changed the American reptile trade, coinciding with a subsequent decline in sales of large, non-native pet reptiles. American pet imports now largely consist of small, docile species such as the crested gecko (*Correlophus ciliatus*) [22].

The python invasion has also funneled large amounts of money into management efforts, such as research conducted by government organizations and the hiring of contractors focused on python removal [18]. This has led to the creation of jobs within academia and the government, as well as an ancillary network of supervisors, equipment manufacturers, vehicle operators, etc. The larger workforce involved in the python management business creates greater public interest, as shown by the emergence of initiatives such as the Florida Python Challenge. The Florida Python Challenge is an annual competition in which members of the public compete to remove the largest, heaviest, and greatest quantity of Burmese pythons from the Greater Everglades ecosystem. Participants in the challenge expressed great concern about the invasion and the importance of raising further public awareness [43].

**Figure 5:**
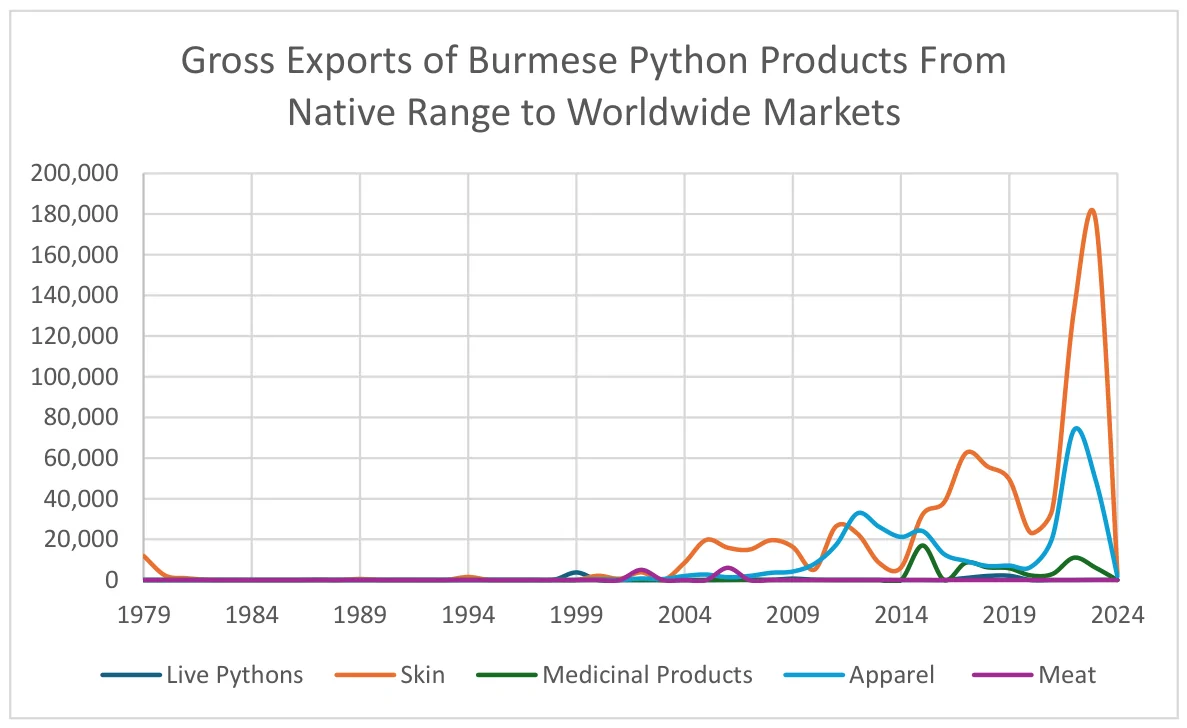
Changes in live Burmese pythons and product exports from 1979 to 2024. These are gross exports and include both direct and indirect trade (including re-exports). For conciseness, exports for each product type were summed. The category, Live Pythons, includes live-hatched pythons and live eggs. Skin products are used for both medical and apparel purposes, so they were given their own category. Medicinal products include oil, bones, and other organs of pythons. Apparel items included processed leather, shoes, jewelry, etc. Meat, like skin, has a variety of uses, though it is most often consumed as food. (Data were extracted from CITES [47]).

Further effects are observed in the sending system due to the flow of pythons from Southeast Asia to South Florida. This feature of telecoupling is called feedback [11]. The trade in live reptiles may be the greatest threat to reptile conservation after habitat loss, and Burmese pythons continue to be used for their skin and medicinal value [44]. Due to this continued consumption, Burmese pythons are currently listed as vulnerable by the IUCN [20]. It is clear, based on Figure 5, that the decline of the Florida market had little overall effect on worldwide exports of live Burmese pythons. Furthermore, the decline of Burmese pythons has continued due to the perseverance of the market [45]. This market remains vital to traders. In Vietnam, snake farming generates an average of $93 USD per month per worker. This helps reduce poverty, underscoring the importance of pythons and other snake species to local people’s finances [46].

## 3. Novel Insights and Lessons Learned from Applying the Telecoupling Framework

The invasion of Burmese pythons has been studied in several ways, but never with the telecoupling framework. This method offers many lessons and insights. These insights include research-oriented insights (identifying gaps in the research), management-oriented insights (the need for flow-centered management of Burmese pythons), and the application of the telecoupling framework to other invasive species.

### 3.1. Identification of Research Gaps

#### 3.1.1. Gaps Related to Each Component of the Telecoupling Framework

Among the sending, receiving, and spillover systems, the largest gap exists in published literature about the sending system. Because their native range is so large and spans multiple countries with complex political relationships, many studies have focused on localized ecological zones such as provinces or national preserves [48,49]. These studies emphasize specific aspects of Burmese python biology. Many studies have focused on movement patterns, habitat use, or potential invasion of other locations, such as the Kinmen Islands (2.1 km off the southern coastline of China) [20,50]. This is important work, but knowledge such as their lifespan in the wild and their abundance in their native range remains unknown [18]. There is even less known about the social and political aspects of the movement of pythons from their native range. The pet trade is poorly understood in general, but even less is known about the Burmese python trade specifically [32]. Existing literature on socioeconomic effects focuses on the skin and leather trade. There is almost nothing about the lives of pet traders or how the pet trade shifted as Burmese pythons established themselves in South Florida.

Research gaps also exist in the spillover and receiving systems, as well as the agents. The spillover system is a vast network comprising shipping locations and the private homes of reptile keepers. The shipping locations are well documented due to government regulations, especially since CITES began monitoring the movement of Burmese pythons in 1975 [18]. Plenty of statistics quantify the number of reptile owners during the python invasion, but there are no figures on the frequency of python escapes. Another difficult spillover opportunity to track is smuggling. Valdez et al. (2021) [22] explain that the probability of an animal being smuggled is directly correlated with its popularity in the marketplace. Burmese pythons have significantly declined in popularity in the US, but as shown by the 2012 case of a Filipino smuggler, undocumented spillover is always possible [51]. Regarding research gaps in the receiving system, much remains unknown about the abundance of pythons in the Greater Everglades ecosystem and the patterns in their daily activities [18].

In terms of flows, the least information exists between the spillover and receiving systems. Even though we know pythons were released or escaped from their owners and made their way into the wild, the timing requires further research. They likely established themselves after the release of a small number of individuals sometime before 1985, but this is a broad time window [27]. Similarly, the flow of pythons further into South Florida requires more research. The inaccessibility of the habitat and the area’s low human population have made it difficult to determine the rate of spread [18].

Fortunately, most of the causes of the Burmese python invasion are well studied. Small gaps remain regarding the number of individuals released and the exact timing, but these have been previously outlined. Overall, the causes have been well researched, but the effects have not been fully examined. The full extent of the trophic cascades, mammalian declines, the spread of parasites, and changes in vegetation dynamics remains to be seen [18]. This is not a result of negligence in academia but a natural consequence of time. Serious research into the Burmese python invasion began in the early 2000s, despite reports dating back to the 20th century [18]. The ecological effects will continue to be studied, and the impacts of Burmese pythons in South Florida will be more clearly defined.

As with the sending system and agents, significant research gaps remain regarding feedback. There is little evidence of how the sending system was affected by the receiving system.

A major strength of the telecoupling analysis is that it shows the research gaps are not independent or equally important. We prioritize the research gaps according to their ecological significance, management relevance, and contribution to advancing invasion science: native-range ecology and population dynamics; release, escape, and trade pathways (flows); magnitude of ecological impacts in Florida; socioeconomic structure of trade networks; feedback between Florida and Southeast Asia; population abundance and movement in Florida; and stakeholder and governance networks. The highest-priority gaps stem from one overarching deficiency: limited understanding of the sending system and its links to receiving and spillover systems. Conventional reviews focused on Florida would likely prioritize abundance estimates and removal methods within the Greater Everglades ecosystem. In contrast, the telecoupling framework shows that the greatest opportunities to advance both science and management lie upstream, namely in understanding the ecological, socioeconomic, and governance processes operating in Southeast Asia and along the trade pathways connecting regions. This perspective shifts research priorities from local invasion symptoms to the larger network of causes, flows, and feedback that generate and sustain biological invasions.

#### 3.1.2. Cross-Cutting Research Gaps

One benefit of the telecoupling framework is that it can inform new research priorities. In this case, it has shown a need for more research into Southeast Asia.

While research from various Southeast Asian countries examines how large-scale issues such as climate change should inform policy decisions, significant gaps remain in understanding the ecological impacts and responses of private citizens [52].

Southeast Asia has suffered from a lack of knowledge production in Western academia since World War II [53]. Even if a case originates in Southeast Asia, there is a preference to conduct research in the North Atlantic [54]. When research is conducted in the region, most published research on Southeast Asian ecology is led by non-Southeast Asian researchers (67.4%), and only 40.3% involve any Southeast Asian collaborators [55]. Studies focusing on the socioeconomic relationships between humans and the environment are even sparser in these areas, reflecting a lack of a well-rounded understanding of the Southeast Asian region due to Western-centric biases.

Thousands of species are currently classified as invasive worldwide [55]. Some were brought as game species such as the Chinese water deer (*Hydropotes inermis*), a species classified as invasive in the UK. Others were introduced accidentally, such as the eel parasite, *Anguillicola crassus*, currently invading the Great Lakes region of the US and much of Europe [55]. Both species originate from Southeast Asia, and the efficacy of their management efforts is directly related to knowledge from their native ranges. The lack of scholarship on Southeast Asian wildlife and ecology in the West affects global invasive species management, environmental conservation, and cross-cultural understanding.

Lack of information about the Burmese pythons’ sending system affects the overall knowledge of the people in their native range. It has also created gaps in baseline information about their biology that could inform management tools in their invasive range. Characteristics such as their nesting, brooding, and mating behaviors are little understood in the wild [56]. Many studies, particularly from Thailand, document their presence in provinces such as Phetchaburi, Prachuap Khiri Khan, Rayong, and Surat Thani [57,58]. These records help establish the range and habitat types of Burmese pythons, but their behavior and life history remain elusive [59]. Additionally, because Burmese pythons are kept as pets, most of their mating and courtship behaviors are known from observations in captivity. This is studied in both their non-native and native ranges, as in Lyubchenko et al.’s 2025 paper [60]. For example, captive Burmese pythons have mostly viable clutches, with hatchling success rates of 92%; however, wild pythons often lay smaller clutches with several inviable eggs [18]. Another example of this problem is the lack of knowledge of nest site selection. In their native range, Burmese pythons are known to inhabit both urban and rural areas [20]. For example, at Thailand’s Suranaree University of Technology, they were spotted near student dormitories on campus [61]. However, a case from 2025 of a python nesting in concrete pipes is only the second published instance of Burmese pythons using anthropogenic structures as nesting sites [56]. Knowledge of this behavior is important because it allows researchers to construct the life history of Burmese pythons. Without this knowledge, it is difficult to identify the demographic parameters most critical to their population growth, which can inform targeted management efforts [18].

Most of these knowledge gaps have ecological and management implications. Ecologically, the lack of understanding of Burmese python biology in their native range, their abundance in both native and invasive ranges, the number and timeline of released individuals, and the full extent of their ecological damage have strong implications for the ecosystems of their native and invasive ranges. Without understanding these, it is hard to assess the damage of their invasion. Furthermore, these problems have management implications. It is difficult to develop a plan for a problem that is not fully understood. Even once a plan has been developed, it is hard to assess its efficacy without knowing baseline abundance or life history traits. New biological information could help identify biological weaknesses that limit their spread in South Florida and refine current population control methods. Additionally, lack of knowledge about smugglers has ecological and possible policy implications. If there are still Burmese pythons moving into South Florida from international sources or across state lines, they would add fresh individuals to the population and require management strategies to end the flow into South Florida. Ecologically, this would lead to higher python abundance in South Florida, parasite spread, and trophic cascade effects. These research gaps, found within each telecoupling component, significantly affect management techniques and ecological knowledge of the Burmese python invasion.

### 3.2. Flow-Centered Management

The framework can help expand current practices from site-centered management (the focus of management on individual sites) to flow-centered management (the management of flows across sites) [16]. The concept of flow-centered management has already been proposed as a major advantage of telecoupling approaches for migratory species [16] and other sustainability challenges. In the case of pythons, conventional management is focused on detecting and removing pythons where they are already established. The telecoupling analysis instead emphasizes flow-centered management, which seeks to manage the movements of organisms, information, money, and agents across systems. This shift generates several new management insights. (1) Management should target pathways and flows, not only local populations. (2) Prevention and early intervention can be more effective than local eradication. (3) Stakeholder coordination across agencies and jurisdictions becomes a priority. (4) Governance networks should incorporate underrepresented actors, such as international stakeholders. (5) Long-distance trade dynamics and information flows should be considered part of invasive-species management.

To limit further spread of Burmese pythons, population suppression methods are most commonly used and can be effective at local scales with intensive effort. However, because Burmese pythons are rarely detected, once they are consistently observed, they are likely well established in the area. Road surveys are also used to capture and remove pythons. They remove more pythons than other management efforts, but only near roads. Tracking radio-telemetered snakes allows removal of breeding snakes in more remote areas, but it is labor-intensive and does not remove large numbers of individuals [18]. Another detection method is eDNA analysis. This method involves collecting water samples and analyzing them for trace amounts of shed Burmese python DNA. This allows researchers to establish python presence in an area but does not indicate abundance or density [62]. All these methods are designed to persecute pythons at localized sites where they are already established and do little to stop their spread. Flow-centered management efforts would be more effective in a case such as the Burmese python, given the continued expansion of their range. Several proposed tools use a more flow-centered approach. Methods such as in vitro work that could drive sex ratio distortion are promising. Genetic biocontrol could also be used to target and destroy X chromosomes, leading to a male-biased sex ratio in wild populations. Methods like these could help address the inaccessibility of the Greater Everglades ecosystem and the low visual detection rates of Burmese pythons. This is because the population would collapse on its own, without the need for human tracking or removal [18]. These efforts are novel and expensive but could be more effective than the currently used site-based methods.

Most current efforts depend on federal funding; however, researchers agree that long-term, coordinated research is needed to make an observable difference. Research of this nature could help fill gaps in baseline biological information, such as reproductive frequency and age-specific fecundity [18]. More funding is also needed for lab-based research. Previously mentioned genetic biocontrol methods are still in their infancy. The destruction of X chromosomes has been shown to work in the mosquito *Anopheles gambiae* but requires further research and refinement for use in larger organisms such as the Burmese python. In vitro methods are beginning to be applied to Burmese pythons, but they require a greater understanding of the genes and pathways in their genomes [18]. These early efforts indicate a shift toward flow-centered methods, which will likely lead to a more long-term and sustainable solution than current methods.

Another important aspect of flow-centered management is cooperation among agents and an analysis of the stakeholders and their relationships. Through this process, underrepresented stakeholders can be given more importance in the network, creating an integrated approach that can address issues more comprehensively [16]. Government-based stakeholders include the National Park Service, SFWMD, and the United States Geological Survey (USGS). They currently fund and carry out management efforts in the Greater Everglades ecosystem, such as radiotelemetry research and visual surveys aimed at python removal [18]. Other large stakeholders are the federally recognized tribes in the area, such as the Miccosukee Tribe of Indians of Florida [63]. As shown by Figure 2, the Miccosukee Tribe’s reservations border the Big Cypress National Preserve to the east and north, with smaller pieces going as far east as the suburbs of Miami, placing them well within the known range of Burmese pythons [18,64]. Without involving them, there is little flow of information in management efforts. For instance, as of 2019, the Miccosukee tribe was using detector dogs to manage Burmese pythons. This proposed tool has not yet been used by NPS or FWC, and knowledge of its efficacy would be vital to assessing its potential for large-scale use [64]. A flow-centered approach encourages greater cooperation among stakeholders, resulting in a more comprehensive effort [16].

Overall, successful suppression and management of invasive species require comprehensive knowledge of the species’ ecological impacts and abundance and coordinated efforts from all involved stakeholders to effectively manage the invasion [65]. Burmese pythons present a unique set of challenges in their management due to their cryptic nature and data limitations. In terms of management recommendations, the most promising are those that can work around the limitations of visual surveys and enable managers to remove individuals on a larger scale than is currently available. With these in mind, we feel there is the most promise in genetic biocontrol methods, greater cooperation between the various stakeholders, and sustained presence monitoring techniques such as eDNA. Implementing these recommendations would require a policy committed to long-term efforts and funding to support emerging management tools [65].

## 4. Constraints on and Opportunities for Framework Operationalization

Operationalizing the telecoupling framework requires a great interdisciplinary effort that often calls for more labor than a conventional approach. Compared to many other frameworks that integrate human and natural systems, the telecoupling framework is more comprehensive and thus more complex. This is because it focuses on more than one geographic location and seeks to examine the connections between human and natural systems worldwide. Challenges range from limited data availability to difficulties securing funding for long-term, interdisciplinary research [16].

Despite these challenges, researchers are recognizing the benefits of such an integrated and comprehensive framework. Funding has begun to be available for projects using the telecoupling framework from agencies such as the National Science Foundation and Belmont Forum [16]. Advances in technology have also enabled more efficient applications of the telecoupling framework to a variety of topics. A product called the Telecoupling Toolbox has been developed [66]. This mainly consists of an ArcGIS Toolbox v2.3 and a Telecoupling GeoApp v1.1b [67]. An agent-based model called TeleABM also simulates telecoupling processes such as international trade and socioeconomic effects [67,68]. These techniques also align with developments in invasive species research, such as genetic biocontrol tools for population suppression and high-resolution global positioning system (GPS) devices for tracking individual movements [16,18]. Progress in technology aimed at operationalizing the telecoupling framework coincides with advances in invasive species research. This leads to improved techniques for overcoming the challenges posed by the complexity of telecoupling.

There are also challenges in the extent of interdisciplinary cooperation required to conduct telecoupling analyses. The telecoupling framework is effective because it encourages disciplinary researchers to examine the broader context in which their research exists. This is a well-understood benefit and partially explains why the use of the telecoupling framework has increased since it was published in 2013. However, many aspects of the telecoupling framework require analysis by researchers from the various disciplinary perspectives present in each telecoupled system [68]. This can be challenging to coordinate, requiring future efforts to include researchers from multiple disciplines in telecoupling projects. Additionally, the framework’s efficacy is affected by the availability of relevant data. This requires qualitative-quantitative research that can encompass the nuances of telecoupling [69]. This can be achieved through truly interdisciplinary research, such as in anthropology and political ecology, and through deep examination of the causal effects of each component [70]. However, the framework’s flexibility makes it highly applicable to a variety of cases, such as land-use, international trade, and invasive species [68].

Despite the challenges, the telecoupling framework presents a novel approach, particularly to invasive species science. Looking more deeply into the socioeconomic reasons for a telecoupling link between distant factors provides a more complete picture. Much research focuses solely on the biological or direct causes and effects of an invasion, but this does not support future use cases or allow for preventative prediction. From this case alone, we see the underlying “incentives” for an invasion that are not specific to only the Burmese python. For example, we see a link between increased trade between countries, political influences on consumer tastes, and biological invasion. These are not generally connected in the conventional study of the Burmese python invasion. With this knowledge, we can look for more potential invasive species as geopolitics and technology advance and shift, looking for a mix of biological and socioeconomic factors that lead to biological invasions.

## 5. Conclusions

The world has become more interconnected during the 20th century and into the 21st. As this continues, humans increasingly face the introduction of invasive species that often spread across political, cultural, and socioeconomic boundaries. Researching invasive species can no longer focus only on their invasive range; it requires the comprehensive approach offered by the telecoupling framework. The Burmese python began its invasion relatively recently, yet its analysis has shown the vast interconnectivity across places and time periods. This example illustrates the potential of the telecoupling framework to analyze invasive species while acknowledging and exploring the broader human elements of invasion. In addition, applying the telecoupling framework differs from existing Burmese python research by enabling an integrated approach to their invasion that has not been attempted before. Using them as an example, it becomes clear that the telecoupling framework offers an opportunity for invasive-species research to explore interrelationships among the various aspects of invasion from different perspectives.

Compared with conventional invasive-species reviews that primarily focus on ecological processes and patterns within invaded ecosystems, this review, applying the telecoupling framework, generates new insights by explicitly connecting sending, receiving, and spillover systems through flows of organisms, information, capital, people, and policies. In the case of Burmese pythons, this approach reveals previously overlooked relationships among Southeast Asian wildlife trade, media influences, international governance, pet-owner behavior, and ecological impacts in Florida. It also exposes hidden feedback between native and invasive ranges, systematically identifies research gaps across telecoupling components, and highlights the need to shift from site-centered to flow-centered management. By integrating ecological and socioeconomic information across distant coupled human and natural systems, the telecoupling framework provides a more comprehensive understanding of invasion dynamics and identifies management opportunities that are often missed by conventional review approaches.

## Figures and Tables

**Table 1:** Research Achievements and Gaps Regarding Burmese Pythons in the Context of the Telecoupling Framework.

Components of Telecoupling Framework [11]	Telecoupling Components About Burmese Pythons	Specific Telecoupling Components That Have Already Been Studied	Research Gaps
Sending System	Native Range in Southeast Asia	General range and distribution of Burmese pythons, habitat types, and diet	Abundance of Burmese pythons in the native range Burmese python life history Socioeconomic makeup of python-based industry
Receiving System	Invasive range in the Greater Everglades ecosystem	Knowledge of the establishment, rough timeline of establishment, species distribution, and diet	Abundance of Burmese pythons Exact circumstances of invasion
Spillover System	Shipping checkpoints, homes of reptile keepers	Number of reptiles imported into the United States, number of American reptile keepers	Number of US Burmese python owners
Agents	Burmese pythons, Burmese python keepers, pet traders, media organizations, government agencies, researchers	Management efforts, US public opinion of the Burmese python invasion	Pet trader market in the native range
Causes	Increased trade between Southeast Asia and the US, releases, and escapes of pet Burmese pythons	Historical context, reasons pythons may have been released	Degree of pet releases
Effects	Biodiversity loss, spread of parasites, changes in vegetation dynamics	Fewer mammal observations, parasite-spread records	Degree of biodiversity loss Degree of vegetation changes

## Data Availability

For Burmese python occurrence data, see the Global Biodiversity Information Facility occurrence metrics. Imports and exports are available on CITES.
